# P-1370. Clinical Characteristics and Outcomes of Pregnant Patients with Latent Tuberculosis

**DOI:** 10.1093/ofid/ofaf695.1557

**Published:** 2026-01-11

**Authors:** Alana Pinheiro Alves, Denise Araujo, George J Alangaden, Eloy E Ordaya

**Affiliations:** Henry Ford Health, Farmington Hills, MI; Henry Ford Health, Farmington Hills, MI; Henry Ford Health, Farmington Hills, MI; Henry Ford Health, Farmington Hills, MI

## Abstract

**Background:**

Screening and managing pregnant individuals (PI) with latent tuberculosis infection (LTBI) is essential to prevent active tuberculosis (TB) and is CDC-recommended for those at risk. However, U.S. data on LTBI management for PI are limited. This report outlines the characteristics, management and outcomes of PI screened and diagnosed with LTBI.

**Figure 1.**
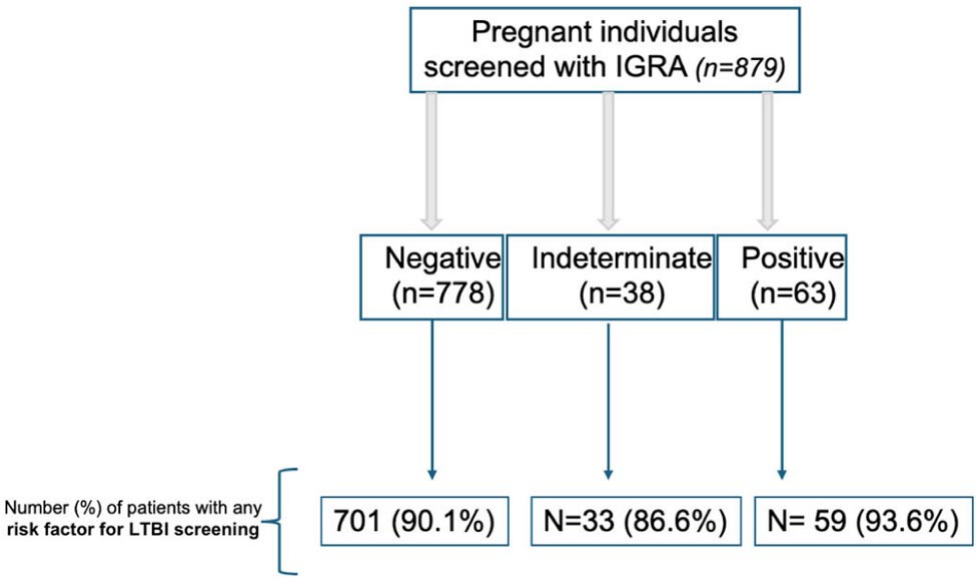
IGRA (Interferon-Gamma Release Assay) test results of pregnant individuals at our center with QuantiFERON-TB Gold Plus (QTF-Plus) or QuantiFERON-TB Gold

**Table 1.**
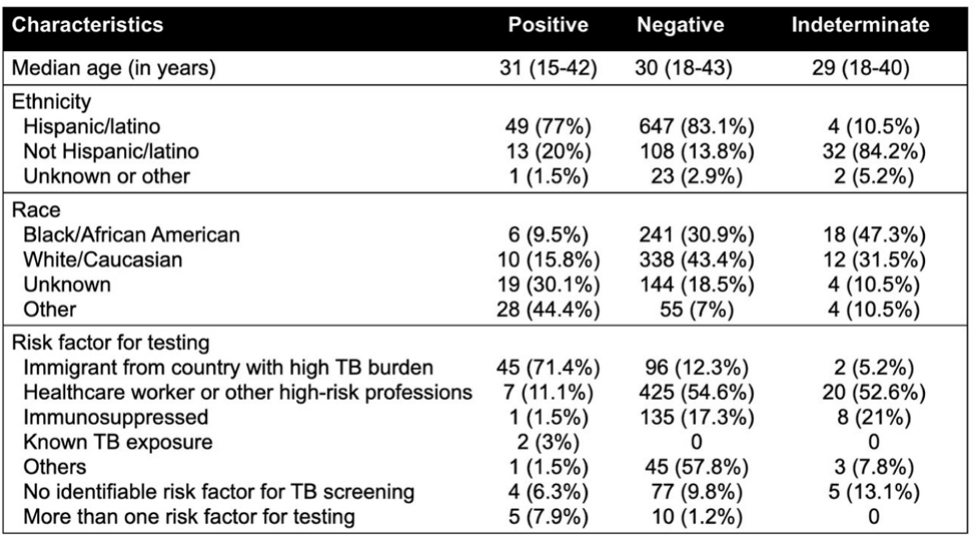
Demographic characteristics and LTBI risk factors of pregnant individuals tested with IGRA at our center

**Methods:**

This retrospective study includes adult (≥18 years) PI screened for LTBI with IGRA at Henry Ford Health between 01/2016 and 12/2024, excluding those previously treated for LTBI or TB.

**Table 2.**
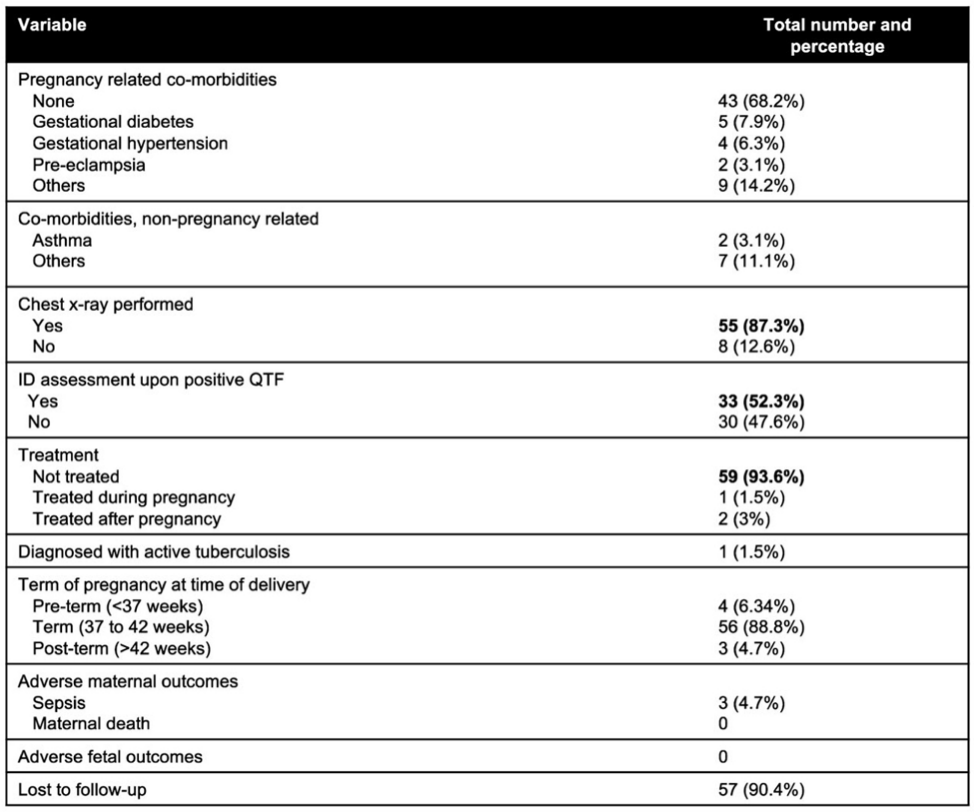
Clinical characteristics and outcomes of pregnant individuals with LTBI (n=63).

**Results:**

A total of 879 PI were screened: 778 (88.5%) negative, 63 (7.1%) positive, and 38 (4.3%) indeterminate (figure 1). The most common risk factor in PI with LTBI was being an immigrant from highly endemic TB countries (71.4%) and being a healthcare worker for those with negative/indeterminate tests, but 10% of all patients were screened without having any risk factor.

For PI with LTBI, median age was 31 years. Only 9 (14.2%) PI had any non-gestational comorbidities. Twenty patients (31.7%) were diagnosed with gestational comorbidities. IGRA was conducted at a median gestational age of 17 weeks. Nearly half of PI with LTBI (33, 52.3%) were evaluated by Infectious Disease (ID). Of these, 30 (91%) were seen before labor, and 87% underwent a chest x-ray (CXR) upon positive screening, identifying one TB case. One patient received therapy during pregnancy, two others (3%) began treatment within the first six months post-partum. Rifampin was used in two patients and isoniazid in one, without significant side effects. One patient was diagnosed with active TB and began treatment during third trimester. After a median follow-up of 382 days (IQR), 90.4% were lost to follow-up. There were no significant adverse maternal or fetal outcomes.

**Conclusion:**

In this study, the leading risk factor prompting LTBI screening was being immigrant from highly endemic countries. Despite no significant adverse maternal or fetal outcomes, only about half PI with LTBI were evaluated by ID, only three (4.8%) received treatment, and most were not followed-up. We identified several missed opportunities in the management of PI with LTBI, which could be leveraged to enhance the cascade of care for this vulnerable population, and further ensure linkage to care for post-partum treatment.

**Disclosures:**

All Authors: No reported disclosures

